# Influence of Ancestry and Molecular Markers on the Severity of Obstructive Sleep Apnea: A Systematic Review and Meta-Analysis

**DOI:** 10.1055/s-0046-1825528

**Published:** 2026-09-02

**Authors:** Luiz J. R. Oliveira, Maria L. R. Guimarães, Luiz De Marco, Nathália S. Guimarães, Luciana Bastos-Rodrigues

**Affiliations:** 1 Department of Nutrition, School of Nursing, Universidade Federal de Minas Gerais (UFMG), Belo Horizonte, Brazil; 2 Laboratory of Technology in Molecular Medicine, School of Medicine, Universidade Federal de Minas Gerais (UFMG), Belo Horizonte, Brazil; 3 Department of Surgery, School of Medicine, Universidade Federal de Minas Gerais (UFMG), Belo Horizonte, Brazil

**Keywords:** sleep apnea obstructive, genetic polymorphism, polymorphism, single nucleotide, ethnic groups, gene-environment interaction, risk factors

## Abstract

Genetic susceptibility to obstructive sleep apnea (OSA) may vary by ancestry. We have conducted a systematic review and random-effects meta-analysis of observational studies (International Prospective Register of Systematic Reviews [PROSPERO] CRD42024548801) to test whether ancestry modifies the associations involving specific genetic polymorphisms and OSA risk and severity. We searched databases until June 2024 for studies examining variants (interleukin-6 (IL6) rs1800795, tumor necrosis factor (TNF) rs1800629, solute carrier family 6 member 4 (serotonin-transporter gene) (SLC6A4) serotonin-transporter-linked polymorphic region (5-HTTLPR)/serotonin transporter intron 2 (VNTR) (STin2), 5-hydroxytryptamine (serotonin) receptor 2A gene (HTR2A) rs9526240, leptin receptor (LEPR) rs3790435) and pooled odds ratios (ORs) with 95% confidence interval (CIs); ancestry-specific effects were explored. Five studies included 3,606 participants. The overall association showed an OR = 1.25 (95%CI: 0.85–1.86; I ^2^ = 76%). By ancestry, Brazilian participants of European descent demonstrated higher risk (OR = 2.80; 95%CI: 1.11–7.08), while those of West-African ancestry showed protection (OR = 0.26; 95%CI: 0.09–0.74). Genetic associations with OSA differ significantly by ancestry, supporting ancestry-informed study designs and larger multiethnic cohorts for personalized OSA management.

## Introduction

Obstructive sleep apnea (OSA), also called *obstructive sleep apnea syndrome* (OSAS) in the older literature, is characterized by repeated airflow blockages during sleep, leading to hypoxemia and sleep disruption. 1 2 It associates with metabolic syndrome and cardiovascular complications including hypertension, coronary artery disease, stroke, and heart failure, affecting more than 1 billion individuals worldwide with variations according to age, sex, and ethnicity. 3 4

Genomic research reveals genetic contributions to OSA through single-nucleotide polymorphisms (SNPs) affecting vulnerability and severity. 5 6 7 8 Potential pathways include metabolic, inflammatory, and circadian mechanisms linking OSA to cardiometabolic outcomes. 9 Variants in interleukin-6 (IL6), tumor necrosis factor-Alpha (TNF-α), PTGER3, LPAR1, C-reactive protein (CRP), glial cell line-derived neurotrophic factor (GDNF), and serotonin transporter have shown associations. 10 11 12 13

Ancestry modifies genetic risk, with Chinese Han, Hispanic/Latino, and African American populations showing variable genetic signal intensity for OSA. 6 14 These differences impact OSA prevalence, severity, and clinical outcomes. 15 16 17 Despite ongoing genome-wide association studies (GWASs) identifying OSA loci, relationships between specific polymorphisms and ancestral backgrounds remain unclear. 5 6 14

The current systematic review and meta-analysis examines whether ancestry modifies associations involving prespecified polymorphisms (interleukin-6 (IL6) rs1800795, tumor necrosis factor (TNF) rs1800629, solute carrier family 6 member 4 (serotonin-transporter gene) (SLC6A4)serotonin-transporter-linked polymorphic region (5-HTTLPR)/serotonin transporter intron 2 (VNTR) (STin2), 5-hydroxytryptamine (serotonin) receptor 2A gene (HTR2A) rs9526240, and leptin receptor (LEPR) rs3790435) and OSA presence/severity. We hypothesized ancestry–variant interactions and explored sex and phenotype definitions as heterogeneity sources. 12 13

## Materials and Methods

### Study Design and Registration

**Fig. 1:**
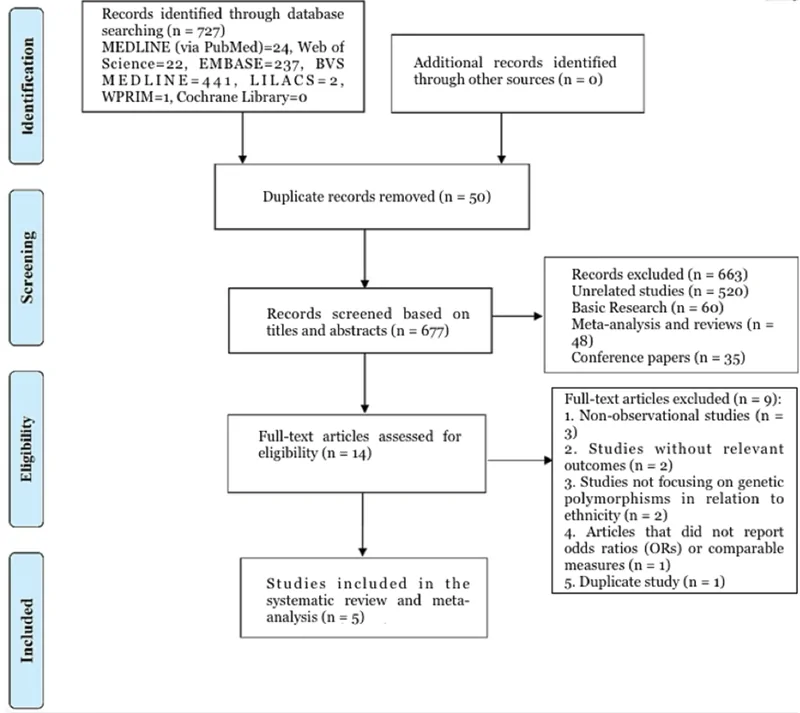
**Preferred Reporting Items for Systematic Reviews and Meta-Analyses (PRISMA) flow diagram of study selection** . The boxes show the number of records identified, screened, excluded and finally included in the quantitative meta-analysis.

We followed the Cochrane Handbook and reported according to the Preferred Reporting Items for Systematic Reviews and Meta-Analyses (PRISMA) statement. 18 The protocol was registered in the International Prospective Register of Systematic Reviews (PROSPERO; CRD42024548801) prior to data extraction. The PRISMA flow diagram for study selection is presented in Fig. 1 .

### PECO

P (population) – Adults (aged ≥ 18 years) evaluated for OSA;
E (exposure) – prespecified genetic polymorphisms (IL6 rs1800795; TNF rs1800629; SLC6A4 5-HTTLPR/STin2; HTR2A rs9526240; and LEPR rs3790435);
C (comparison) – genotype/allele contrasts within ancestry strata and/or across ancestries;
O (outcome) – OSA presence and severity (based on the apnea-hypopnea index [AHI]).

Data standardization: per-allele, odds ratios (ORs) were extracted preferentially and effect alleles were harmonized; when multiple genetic models were reported, we prioritized allelic models, followed by dominant models. Allele-frequency plausibility (prespecified): for each SNP, control Minor Allele Frequency (MAFs) were to be compared with ancestry-matched Genome Aggregation Database (gnomAD) panels; if |ΔMAF| > 10 percentage points, we would downgrade selection bias in the Newcastle–Ottawa Scale (NOS) and plan a sensitivity analysis excluding such studies. Given the small, heterogeneous evidence base and incomplete MAF reporting in some studies, this exclusion trigger was not uniformly assessable, and no MAF-triggered exclusions were implemented.

Regarding terminology, (the definitions of ancestry, ethnicity, and race), throughout the current review, we use *ancestry* to refer to genomically- or geographically-inferred population origin — an objective, measurable construct captured by tools such as ancestry-informative markers (AIMs) or principal-component analysis of genotype data, following established frameworks in genetic epidemiology. 15 We use *ethnicity* to refer to self-reported sociocultural identity based on shared culture, language, or national origin, which is subjective and may not correspond to genomic ancestry. We retain the term *race* only when it appears in the original terminology of a cited primary study (such as *African American* , *European American* , as used by Larkin et al. 6 ); in our own synthesis, we use *ancestry* as the overarching umbrella term. This distinction is consequential: the Guindalini et al. 19 study quantified ancestry using AIMs, whereas the Larkin et al. 6 20 studies used self-reported racial classification, and these approaches capture partially-overlapping but non-identical constructs. When genomic and self-reported classifications converge, we refer to ancestral groups by their geographic descriptor (such as *West-African ancestry* , *European ancestry* ); when studies used racial labels, we preserve those labels in study-specific descriptions.

### Outcome Measures

The primary outcome was the association between genetic polymorphisms and OSA presence (diagnostic status), and the secondary outcomes were the associations with OSA severity (AHI-based thresholds) and ancestry-specific effects. We extracted or computed ORs with 95%VIs.

### Search Strategy

We conducted a broad literature review on the MEDLINE (via PubMed), EMBASE, Web of Science, LILACS, WPRIM, and Cochrane Library electronic databases for studies published in English, Spanish or Portuguese. The search included several keywords related to OSA and genetic polymorphisms, such as *Obstructive Sleep Apnea* and its variations, *Genetic Polymorphisms* , *Single Nucleotide Polymorphism* , and terms related to ethnicity and ancestry. The allele frequencies for each SNP were cross-checked in the gnomAD, version 4 (global and ancestry-specific panels). to ensure plausibility of minor-allele frequencies reported by primary studies.

The descriptors used were from recognized controlled vocabularies such as the Medical Subject Headiungs (MeSH), Health Sciences Descriptors, and Emtree. The search strategies for each database are in Appendix A .

### Eligibility Criteria

The inclusion criteria for the current review and meta-analysis were designed to match the main aim of exploring the effect of ethnicity and genetic markers on OSA. Studies were included if they met the following criteria: Adults aged ≥ 18 years with diagnosed OSA were the population of interest. We only included studies that looked at genetic inheritance and OSA, with a focus on occurrence and degree of severity of the condition. The included studies had to provide comparisons across different ethnic groups or among individuals with different genetic profiles so we could look at genetic susceptibility to OSA. The primary outcome was the reporting of ORs for genetic polymorphisms associated with OSA, with the AHI as the main outcome measure for the severity of the condition. All included studies reported that genotype counts conformed to the Hardy–Weinberg equilibrium ( *p* > 0.05); linkage disequilibrium among targeted SNPs was not relevant because each study analyzed independent variants.

Study design: We included observational studies such as cohort, case-control and cross-sectional studies that looked at genetic variations and ethnicity in OSA. This broad inclusion criterion was to capture all genetic predispositions across different populations.

Exclusion criteria: To focus on adult populations, we excluded studies that involved children or adolescents. We also excluded articles that did not separate data by ethnicity or race as this did not enable us to look at genetic and ethnicity variations that affect OSA degree of severity. We also excluded studies that did not address the genetic basis of OSA and those that did not report ORs, which is necessary for the meta-analytical synthesis of the genetic associations.

### Data Extraction

Data extraction was performed using a standard template by two reviewers independently. When there were any differences in opinion, a third party was called upon for arbitration. The extracted information comprised study details such as author(s), publication year, population characteristics, genes investigated, ethnic background, sample size, as well as ORs and their respective CIs relating genetic features to OSA degree of severity. When numeric details were incomplete or unclear, we contacted the corresponding authors of the primary studies by e-mail and requested the missing information.

### Primary versus Exploratory Variants

Primary variants: IL6 rs1800795 20 ; TNF rs1800629 10 ; SLC6A4 5-HTTLPR/STin2 9 ; HTR2A rs9526240 6 ; and LEPR rs3790435 21 (prespecified from previous literature and data availability). Exploratory variants were summarized without confirmatory testing. Given the small and sparse evidence base, we did not apply multiplicity-adjusted hypothesis testing; effect sizes with 95 CIs are presented, and subgroup signals are interpreted as hypothesis-generating.

### Risk of Bias Assessment

Two reviewers independently assessed risk of bias using the NOS, resolving disagreements by consensus (and a third reviewer when needed). We summarize concerns at the domain level (selection, comparability, exposure/outcome). We did not apply the Grading of Recommendations Assessment, Development, and Evaluation (GRADE) approach because all included studies 6 9 19 20 21 were observational, and the small evidence base (k = 5) limits meaningful rating; accordingly, the overall certainty of evidence is low.

### Statistical Analysis

For the statistical analysis, we used the R (R Foundation for Statistical Computing) software, version 4.2.2. Random-effects models (DerSimonian–Laird) pooled study-level ORs. Heterogeneity was quantified with I ^2^ and Q. Genetic effect models (allelic and, when available, dominant) were harmonized; if multiple models were reported, the allelic model was prioritized. When a cell count was zero, a 0.5 continuity correction was applied.

We prespecified qualitative labels for pooled effects: *small* (OR: 1.00–1.10), *modest* (OR > 1.10–1.50), *moderate* (OR > 1.50–2.00), and *large* (OR > 2.00); analogous thresholds were applied for OR < 1.00 by symmetry. The labels are descriptive and do not override CIs. Planned meta-regression (moderators: ancestry, sex, AHI threshold, desaturation criterion, and geographic region) was to be performed when ≥ 10 effects were available; this threshold was not met, so no meta-regression was fitted or reported.

Rationale for global candidate-variant synthesis: Although the prespecified polymorphisms (IL6 rs1800795, TNF rs1800629, SLC6A4 5-HTTLPR/STin2, HTR2A rs9526240, and LEPR rs3790435) operate through distinct biological pathways—inflammatory, serotonergic, and metabolic—, a global pooled estimate was computed as a candidate-variant synthesis, following the approach used in umbrella analyses of genetic-susceptibility loci. This global estimate is intended solely as a descriptive summary of the overall direction and magnitude of genetic associations with OSA across the included studies; it does not imply a single shared biological mechanism. The primary analytic focus of the present review is on variant-specific and ancestry-specific subgroup estimates, which are the interpretively-meaningful results. The global pooled OR should therefore be read as a contextual reference point, and the anticipated high heterogeneity (attributable in part to the structural diversity of the pooled variants) is an expected property of this design rather than an analytical artifact.

We did not formally assess the certainty of evidence using the GRADE approach, as all included studies were observational genetic-association studies, which begin at low certainty in GRADE by default and were too few and heterogeneous for meaningful upgrading or downgrading.

## Results

### Study Selection

In June 2024, a comprehensive database search conducted using predefined search criteria across PubMed, EMBASE, Web of Science, LILACS, WPRIM, and Cochrane Library identified 727 records. After removing 50 duplicates, 677 studies remained for title and abstract screening. Based on the preestablished inclusion and exclusion criteria, two independent reviewers assessed these studies. The conflicts between reviewers were resolved by a third reviewer when necessary. As a result of the initial screening phase, 663 studies were excluded due to the exclusion criteria ( Appendix B ), leaving 14 articles for eligibility assessment. Ultimately, five studies 6 9 19 20 21 met the full eligibility criteria and were included in the systematic review and meta-analysis. A detailed PRISMA flowchart of the study selection process can be found in Fig. 1 .

A total of 3,606 participants from a variety of ancestry groups, including Chinese Han (25.3%), African Americans (27.4%), European Americans (19.2%), and admixed populations from Brazil (28%) with European, West-African, and Native American ancestry, are included in the 5 studies. These investigations assessed various sets of genetic markers connected to the occurrence of OSA, offering a thorough analysis of genetic variants linked to the condition. The *LEPR* , *serotonin transporter* ( *5-HTT* ), *GDNF, IL-6* , 20 and other inflammatory indicators including *TNF-α* and *CRP* were among the genes that were searched for in the study. The main outcome measure used in all the research to indicate the occurrence and degree of severity of OSA was the AHI. The main statistical method to evaluate the connection between genetic variations and OSA outcomes was thought to be the ORs. A detailed review of the sample sizes, demographics, and genes analyzed is provided in Table 1 .

**Table 1:** Characteristics of studies investigating the association between genetic polymorphisms and OSA across different ethnic populations

Study	Country	Population Size (n)	Age in years (mean and/or range)	Male sex (%)	Genes analyzed	OSA measurement	Covariates	Study design
Larkin et al., 20 2010b	United States	African Americans: 259	51.1 ± 13.0 years	58%	*IL6* (polymorphisms: *IL6–* 1111, *IL6–* 1510, *IL6–* 2892, *IL6–* 3572, *IL6–* 6021, *IL6–* 7592)	AHI ≥ 15 events/hour or current treatment with CPAP; AHI < 5 events/hour for controls	Age, sex, BMI	Case-control
Guindalini et al., 2010 19	Brazil	Adults from São Paulo (admixed ancestry, including European, West African, and Native American): 1,010	Mean: 42.4 years	44.30%	*31 ancestry-informative markers (AIMs)*	Polysomnography (AHI > 5 events/hour with symptoms or ≥ 15 events/hour regardless of symptoms)	Sex, age, BMI, socioeconomic status	Cross-sectional
Li et al., 21 2019	China	Chinese Han: 322	55.3 ± 10.8	85.80%	*LEPR*	Polysomnography, AHI ≥ 5 events/hour	Age, sex, BMI, triglycerides, TC, LDL-C, HDL-C, FBG, smoking status, and alcohol use	Case-control
Yue et al., 9 2008	China	Chinese Han: 592 (254 OSA patients, 338 controls)	45.2 (OSA), 43.2 (controls)	86.6% (OSA), 86.1% (controls)	*Serotonin transporter (5-HTT) gene polymorphisms (5-HTTLPR and STin2.VNTR)*	Polysomnography with AHI > 5 events/hour	Excluded subjects with psychiatric disorders, serotonergic medication, and conditions affecting serotonin levels	Case-control
Larkin et al., 6 2010a	United States	European Americans (694) and African Americans (729)	European Americans: mean: 38.8 (range 3–81); African Americans: mean: 36.4 (range 3–82)	European Americans: 47%; African Americans: 42%	*CRP, GDNF, HTR2A*	AHI ≥ 15 events/hour	Age, age-squared, sex, BMI, racial admixture	Cross-sectional

**Abbreviations:** 5-HTT, serotonin transporter; 5-HTTLPR, serotonin-transporter-linked polymorphic region; AHI, apnea–hypopnea index; BMI, body mass index; CPAP, continuous positive airway pressure; CRP, C-reactive protein; FBG, fasting blood glucose; GDNF, glial cell line-derived neurotrophic factor; HDL-C, high-density lipoprotein cholesterol; HTR2A, 5-hydroxytryptamine (serotonin) receptor 2A gene; IL6, interleukin-6; LDL-C, low-density lipoprotein cholesterol; LEPR, leptin receptor; OSA, obstructive sleep apnea; STin2, serotonin transporter intron 2; TC, total cholesterol; VNTR, variable number tandem repeat.

### Meta-Analysis

**Fig. 2:**
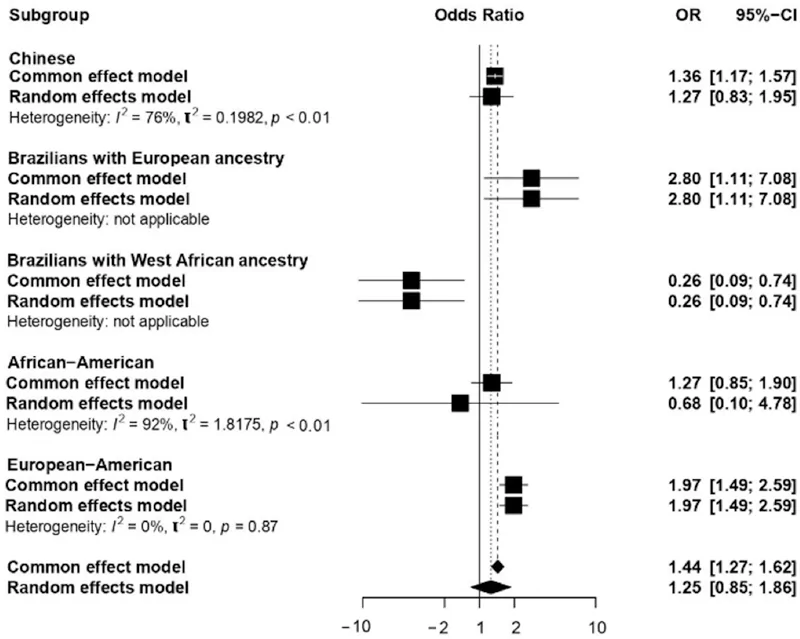
**Pooled odds ratios (ORs) for the association between genetic polymorphisms and obstructive sleep apnea, with subgroup analysis by ancestry.** The squares indicate study-specific ORs sized by inverse-variance weight; the horizontal lines show 95%CIs; the diamonds depict random-effects (RE) pooled estimates for each ancestry group and for all studies combined. **Abbreviation:** OSA, obstructive sleep apnea.

The pooled ORs for the relationship between genetic polymorphisms and the occurrence of OSA were estimated using a random-effects model, reflecting the heterogeneity among the studies (I ^2^ = 76%; *p* < 0.01); Fig. 2 displays study-specific estimates and weights for each ancestry subgroup. A modest, non-significant link between specific genetic variants and the occurrence of OSA was indicated by the overall pooled OR of 1.25 (95%CI: 0.85–1.86), although the large CIs raise doubts about the strength of this relationship without statistical significance. This global estimate represents a candidate-variant synthesis across biologically-heterogeneous polymorphisms and should be interpreted as a descriptive summary of overall effect direction rather than as evidence of a single shared genetic mechanism; the variant-specific and ancestry-stratified subgroup analyses presented below constitute the primary focus of the current review.

Subgroup analyses including several ancestry groups showed a notable variation in genetic correlations. Brazilians of European ancestry, for example, showed a substantially higher OR, of 2.80 (95%CI: 1.11–7.08), as shown in the forest plots ( Fig. 2 ), indicating a genetic propensity to OSA degree of severity in this group. Brazilians of West-African ancestry, on the other hand, had a significantly reduced OR, of 0.26 (95%CI: 0.09–0.74), suggesting that this population may be genetically protected against severe OSA. The complexity of genetic factors regarding OSA across diverse populations is highlighted by this ancestry-related variation. These contrasts warrant cautious interpretation given the small number of contributing studies.

Analysis of Asian populations revealed that Chinese Han subjects with the LEPR rs3790435 CC genotype demonstrated substantial protection against OSA compared with carriers of other genotypes. In one key study by Li et al., 21 which included 322 Chinese Han individuals, subjects with the CC genotype had a markedly-lower risk of developing OSA compared with those with TT/TC genotypes (OR: 0.462; 95% CI: 0.250–0.854; *p* = 0.014). Among obese subjects specifically, those with the CC genotype maintained this protective effect (OR: 0.191; 95%CI: 0.041–0.878; *p* = 0.033), suggesting the variant's protective influence persists even in the presence of this major risk factor. 21

The analysis of serotonergic-pathway genes revealed substantial associations with OSA risk. Variants in the *HTR2A* gene, particularly the rs9526240 polymorphism, demonstrated a strong association with increased OSA susceptibility in African Americans (OR: 2.1; 95%CI: 1.5–2.9). 6 Similarly, polymorphisms in the *5-HTT* gene, including 5-HTTLPR and STin2.VNTR, were associated with OSA in Chinese Han populations. Notably, the 10-repeat allele of STin2.VNTR showed a substantial association with OSA in males (OR: 1.94; 95%CI: 1.26–3.00). These findings highlight the role of serotonergic mechanisms in upper airway control and the potential for genetic variants in these pathways to influence OSA susceptibility across diverse populations. 6 9

### Subgroup and Sensitivity Analyses

**Fig. 3:**
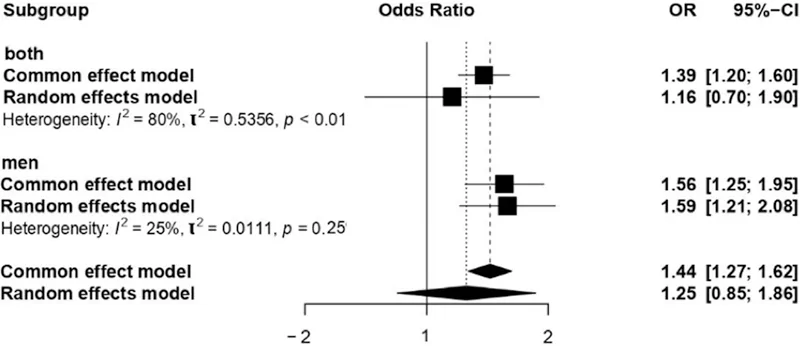
**Genetic association with OSA stratified by sex** . Forest plot displays study-specific ORs and 95%CIs for mixed-sex cohorts and for male populations only; pooled effects are shown as diamonds.

Other subgroup analyses were conducted to understand the differences in OSA occurrence based on sex, ethnicity, and clinical OSA characteristics. Fig. 3 shows that the relationship involving genetic polymorphisms and OSA was strong in male participants (OR: 1.59; 95%CI: 1.21–2.08), compared with studies including both sexes (OR: 1.16; 95%CI: 0.70–1.90).

**Fig. 4:**
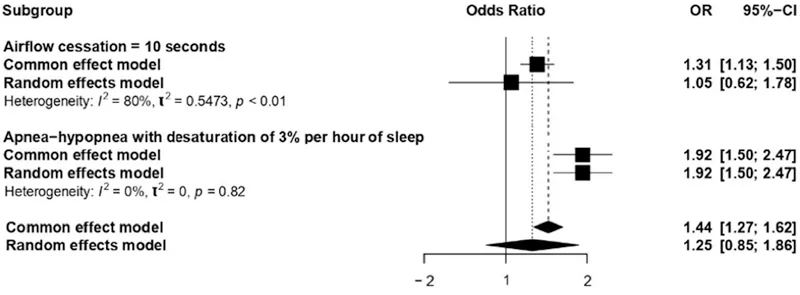
**Forest plot of genetic associations with OSA by diagnostic criteria, comparing airflow cessation and apnea-hypopnea events** . The analysis shows pooled estimates using RE models with ORs and 95%CIs.

**Fig. 5:**
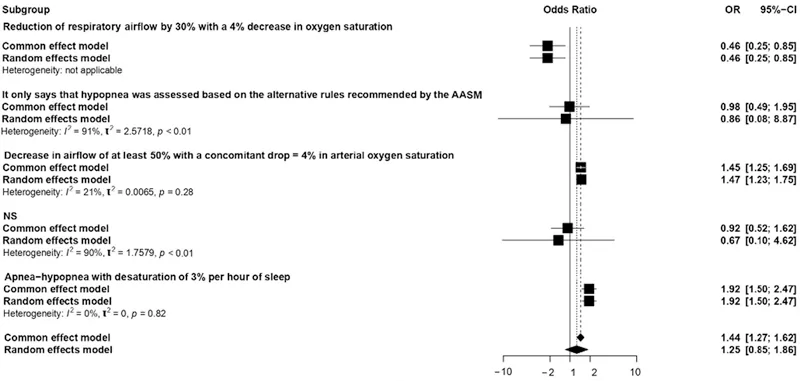
Pooled genetic associations across hypopnea/desaturation phenotype definitions for OSA. The diamonds represent random-effects (RE) pooled ORs (95% CIs) for OSA defined under alternative hypopnea-scoring and oxygen-desaturation criteria, including an apnea–hypopnea definition requiring ≥3% desaturation (OR 1.92; 95%CI 1.50–2.47). RE, random effects; OR, odds ratio; CI, confidence interval; OSA, obstructive sleep apnea.

The results indicate that the genetic associations with OSA vary based on the definitions used for apnea-hypopnea events. Fig. 4 presents a subgroup analysis comparing airflow cessation and apnea-hypopnea event definitions. Using the criterion of airflow cessation ≥ 10 seconds, the pooled association was OR: 1.05 (95%CI: 0.62–1.78; I ^2^ = 80%). Using AHI with ≥ 3% desaturation definitions, the association strengthened to OR: 1.92 (95%CI: 1.50–2.47; I ^2^ = 0%). These differences, further illustrated in Fig. 5 , suggest that phenotype definitions materially influence observed genetic effects.

**Fig. 6:**
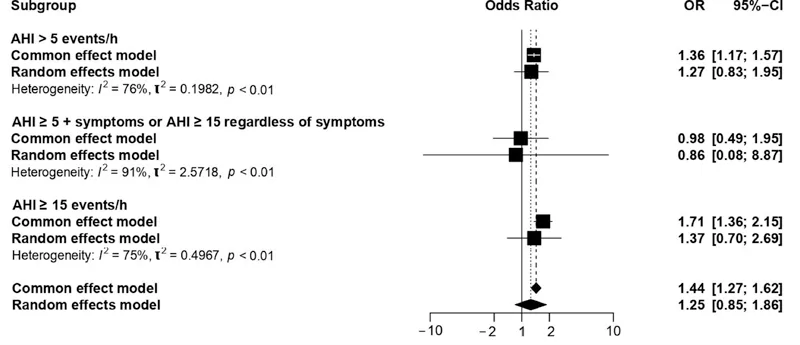
Pooled genetic associations across diagnostic thresholds for OSA. The diamonds represent RE pooled ORs (95% CIs) for studies using apnea -hypopnea index (AHI) > 5 events/h, AHI ≥ 15 events/h, or a combined criterion (AHI ≥ 5 with symptoms/AHI ≥ 15 irrespective of symptoms). AHI, apnea-hypopnea index; OR, odds ratio; CI, confidence interval; OSA, obstructive sleep apnea.

The subgroup analysis based on varying diagnostic criteria revealed distinct genetic associations with OSA. Studies employing a diagnostic threshold of AHI > 5 events/hour reported a pooled OR of 1.27 (95%CI: 0.83–1.95), with notable heterogeneity (I ^2^ = 76%; *p* < 0.01). In studies using more stringent criteria, such as AHI ≥ 15 events/hour 6 20 , the genetic association with OSA was stronger, yielding an OR of 1.37 (95%CI: 0.70–2.69) and moderate heterogeneity (I ^2^ = 75%; *p* < 0.01). Conversely, when the diagnostic criteria included both AHI ≥ 5 with symptoms or AHI ≥ 15 regardless of symptoms, the pooled OR dropped to 0.86 (95%CI: 0.08–8.87), showing the weakest association and the highest heterogeneity (I ^2^ = 91%; *p* < 0.01). These findings, summarized in Fig. 6 , also highlight the critical influence of phenotype definitions on the strength and consistency of genetic associations in OSA research.

## Discussion

The findings of the present study indicate possible ancestry-related differences in variant–OSA associations; however, the overall pooled effect was non-significant, with substantial heterogeneity and few contributing studies. Observational designs introduce risks of confounding and population stratification. Conclusions are hypothesis-generating; clinical screening recommendations are premature.

The meta-analysis identified a modest overall association between genetic variants and OSA (OR: 1.25; 95%CI: 0.85–1.86), but with substantial heterogeneity (I ^2^ = 76%; *p* < 0.01) that appears largely attributable to ancestry-related differences. Notably, we found striking ancestry-related variations in genetic susceptibility. Brazilians of European ancestry showed substantially-increased risk (OR: 2.80; 95%CI: 1.11–7.08), while those of West-African ancestry demonstrated protection against OSA (OR: 0.26; 95%CI: 0.09–0.74). These findings align with those of previous admixture mapping studies 14 in Hispanic/Latino populations that have identified differential genetic effects based on ancestral background. It should be acknowledged that the substantial heterogeneity in the global pooled estimate reflects not only ancestry-related variation, but also the structural diversity of the pooled polymorphisms themselves: combining variants from distinct biological pathways—inflammatory (IL6, TNF, CRP), serotonergic (HTR2A, SLC6A4), and metabolic (LEPR)—is an inherent feature of candidate-gene umbrella analyses with sparse evidence bases, and the resulting I ^2^ should be interpreted in this context rather than as evidence of methodological inconsistency.

The genetic associations with OSA showed a distinct pattern in sex-stratified analyses ( Fig. 3 ), with stronger effects observed in male (OR: 1.59; 95%CI: 1.21–2.08) compared with mixed-sex populations (OR: 1.16; 95%CI: 0.70–1.90). This difference supports existing clinical evidence 5 indicating sexual dimorphism in OSA presentation and progression.

The SNP panel examined in the current review—IL6 rs1800795, TNF rs1800629, SLC6A4 5-HTTLPR/STin2, HTR2A rs9526240, and LEPR rs3790435—was intentionally limited to prespecified candidate variants selected on the basis of prior mechanistic evidence linking inflammatory, serotonergic, and metabolic pathways to OSA pathophysiology. 6 9 12 This approach is complementary to, but distinct in scope from, GWASs. Recent large-scale GWAS have substantially expanded the catalogue of OSA-associated loci: the Million Veteran Program 5 identified genomic regions with sex-heterogeneous effects across diverse ancestry groups, Strausz et al. 22 identified loci near SLC9A8, GABBR1, and LRP1 in a European-ancestry cohort with evidence for cardiometabolic coregulation, and Xu et al. 23 characterized multiancestry OSA loci with explicit ancestry-stratified analyses revealing population-specific genetic architecture. None of these GWAS-derived variants were captured by our prespecified candidate panel, and this constitutes a recognized scope limitation: the present review is not designed as a comprehensive GWAS synthesis, but rather as a focused interrogation of specific biological pathways across ancestral backgrounds. The strength of this design lies precisely in the mechanistic specificity it affords—enabling the examination of how variants in defined pathways demonstrate differential effects by ancestry—, a resolution that is often diluted in hypothesis-free genome-wide approaches. 14 24 Consequently, the candidate-gene and GWAS frameworks should be viewed as addressing complementary scientific questions, and future meta-analyses integrating GWAS-derived variants with ancestry-stratified designs will be essential to build on these findings.

The substantial heterogeneity observed in the analyses (I ^2^ = 80% for mixed-sex populations and 25% for male populations) underscores the complexity of genetic contributions to OSA and highlights the necessity for future studies to adopt standardized methodologies and carefully account for population-specific factors. These results emphasize the importance of considering sex as a biological variable in genetic research and suggest that tailored interventions based on sex may optimize OSA management and outcomes.

The analysis of varying diagnostic criteria for OSA, as presented in Fig. 6 , demonstrates that the genetic association with OSA is most pronounced when applying a threshold of AHI ≥ 15 events/hour (random-effects OR: 1.37; 95%CI: 0.70–2.69; I ^2^ = 75%). This stronger association suggests that genetic factors may exert a greater influence on more severe cases of the condition. Conversely, studies using less stringent diagnostic criteria, such as AHI > 5 events/hour 9 21 , reported weaker associations (OR: 1.27; 95%CI: 0.83–1.95; I ^2^ = 76%), while those using combined criteria (AHI ≥ 5 with symptoms or AHI ≥ 15 regardless of symptoms) 19 yielded the weakest association (OR: 0.86; 95%CI: 0.08–8.87) and the highest heterogeneity (I ^2^ = 91%; *p* < 0.01). These findings highlight the importance of diagnostic standardization and suggest that more severe disease phenotypes may be more strongly influenced by genetic factors. This aligns with evidence from twin studies, 25 26 which indicate greater heritability for severe forms of OSA compared with mild or moderate cases. The observed heterogeneity across diagnostic thresholds underscores the complexity of genetic contributions to OSA and emphasizes the need for consistency in phenotype definitions to improve the reliability of genetic association studies. 25 26

Our analysis of apnea-hypopnea event subgroups, as depicted in Fig. 5 , underscores the importance of standardized diagnostic criteria in genetic studies of OSA. The findings revealed a genetic association when using AHI criteria that included a 3% oxygen desaturation requirement (OR: 1.92; 95%CI: 1.50–2.47). This association suggests that genetic factors may have a greater impact on phenotypes defined by more stringent clinical thresholds, which likely represent more severe disease presentations. These results align with those of previous research 12 emphasizing the significance of phenotype definitions in elucidating genetic underpinnings of OSA. Such standardization may also help address the heterogeneity observed in earlier studies 13 17 , facilitating a clearer understanding of how genetic variations contribute to the pathophysiology of OSA. Future research should prioritize the use of consistent diagnostic criteria to enhance the comparability and reliability of genetic findings in this field. 1 27

Our results should be read with care. Several ancestry groups were represented by few studies, which limits firm, population-specific inferences.

A potential source of non-independence deserves explicit acknowledgment: Larkin et al. 2010a 6 and Larkin et al. 2010b 20 both recruited from the Cleveland Family Study (CFS), and the smaller 2010b case-control sample represents a subset of the larger 2010a cohort. Because the two studies examined entirely different genetic variants—HTR2A, CRP, and GDNF in 2010a, and IL6 polymorphisms in 2010b—, their effect estimates are biologically independent, even where participants overlap. Both studies were retained to preserve the full scope of the candidate-variant evidence base; however, we conducted a sensitivity analysis excluding Larkin 2010b from the global pooled estimate and the African American subgroup, which did not materially alter the pooled OR nor heterogeneity estimates, supporting the robustness of the primary results.

Furthermore, restricting SNP selection to candidate variants based on prior mechanistic evidence means that genetic loci identified through recent large-scale GWASs— including variants near SLC9A8, GABBR1, and other regions with plausible roles in OSA pathophysiology—were not captured by the current analysis; future systematic reviews should incorporate GWAS-derived variants, with particular attention to those demonstrating ancestry-specific effect sizes, to provide a more comprehensive picture of the genetic architecture of OSA. 22 23

Case definitions and severity cut-offs for OSA varied across reports, complicating pooled estimates, and some cohorts relied on self-reported racial or ethnic classification rather than genomically-inferred ancestry—notably the Larkin et al. 6 20 studies, which categorized participants as *African American* or *European American* based on self-report—raising the possibility of ancestral misclassification that could attenuate or distort estimated genetic associations, particularly for admixed individuals whose genomic ancestry may not align with their self-identified racial group. 12 15 24 We could not uniformly verify control MAFs against gnomAD because of incomplete reporting; where relevant, we reflected this as downgrades in NOS domains. The most frequent risks of bias were case/control selection, limited adjustment for key confounders (such as adiposity, craniofacial traits), and possible information bias in exposure or outcome ascertainment. With only k = 5 studies 6 9 19 20 21 and substantial heterogeneity, the overall certainty is low, and the findings should be viewed as hypothesis-generating.

Even so, the pattern is consistent with ancestry-modulated genetic effects on OSA, echoing signals observed in Hispanic/Latino, Chinese Han, and African American samples and in large biobank analyses. 5 6 14 If confirmed, risk tools may need ancestry-specific parameters, and pathway-level hints (inflammatory, serotonergic, metabolic) could guide targeted research rather than immediate clinical screening. 12 14 15

The priorities now include larger, multiethnic cohorts, especially in non-European populations, standardized OSA phenotypes and severity thresholds, and explicit tests of gene–environment interplay (obesity, craniofacial structure). 3 13 14 The high heterogeneity we observed (I ^2^ ∼ 75–92%) and stronger signals under stricter diagnostic criteria both argue for careful, harmonized designs going forward. 3 13

In conclusion, the current systematic review and meta-analysis demonstrates the important impact that genetic variants have on the occurrence and degree of severity of OSA. Understanding these genetic predispositions requires an awareness of the role played by ethnicity, with certain ethnic groups exhibiting a higher hereditary risk for severe OSA and others perhaps possessing protective genetic characteristics. These results highlight the need for further investigation across diverse ancestry groups to deepen our understanding of the genetic contributions to this prevalent and clinically-significant sleep disorder, with important implications for the future of ancestry-informed personalized care in OSA management.
