# Supplementary material for: Influence of Ancestry and Molecular Markers on the Severity of Obstructive Sleep Apnea: A Systematic Review and Meta-Analysis
Source: Sleep Sci. 2026 Sep 2;19(2):1–11. doi: 10.1055/s-0046-1825528 (PMC13537835; doi:10.1055/s-0046-1825528)
Supplement: Supplementary file 1 [file 10-1055-s-0046-1825528-s250561.pdf]

Appendix A - Search Strategy.

Databases to be Searched

- PubMed
- EMBASE
- Web of Science
- LILACS
- WPRIM
- Cochrane Library

Search Terms and Strategy

1. **PubMed Search Strategy:**

```
```

#1 “Sleep Apnea Syndromes”[Mesh] OR “Apnea Syndrome, Sleep” OR “Apnea Syndromes, Sleep” OR “Sleep Apnea Syndrome” OR “Apnea, Sleep” OR “Apneas, Sleep” OR “Sleep Apnea” OR “Sleep Apneas” OR “Sleep Hypopnea” OR “Hypopnea, Sleep” OR “Hypopneas, Sleep” OR “Sleep Hypopneas” OR “Hypersomnia with Periodic Respiration” OR “Sleep Apnea, Mixed Central and Obstructive” OR “Mixed Central and Obstructive Sleep Apnea” OR “Sleep Apnea, Mixed” OR “Mixed Sleep Apnea” OR “Mixed Sleep Apneas” OR “Sleep Apneas, Mixed” OR “Sleep-Disordered Breathing” OR “Breathing, Sleep-Disordered” OR “Sleep Disordered Breathing” OR “Sleep Apnea, Obstructive”[Mesh] OR “Apneas, Obstructive Sleep” OR “Obstructive Sleep Apneas” OR “Sleep Apneas, Obstructive” OR “Apnea, Obstructive Sleep” OR “Sleep Apnea Hypopnea Syndrome” OR “Obstructive Sleep Apnea Syndrome” OR “Obstructive Sleep Apnea” OR “Syndrome, Obstructive Sleep Apnea” OR “Syndrome, Sleep Apnea, Obstructive” OR “Sleep Apnea Syndrome, Obstructive” OR “OSAHS” OR “Upper Airway Resistance Sleep Apnea Syndrome” OR “Syndrome, Upper Airway Resistance, Sleep Apnea”
#2 “Racial Groups”[Mesh] OR “Group, Racial” OR “Groups, Racial” OR “Racial Group” OR “Race” OR “Races” OR “Racial Stocks” OR “Racial Stock” OR “Stock, Racial” OR “Stocks, Racial” OR “Continental Population Groups” OR “Continental Population Group” OR “Group, Continental Population” OR “Groups, Continental Population” OR “Population Group, Continental” OR “Population Groups, Continental” OR “Pedigree”[Mesh] OR “Ethnicity”[Mesh] OR “Nationality” OR “Nationalities” OR “Ethnic Groups” OR “Ethnic Group”
#3 “Genetics”[Mesh] OR “Polymorphism, Genetic”[Mesh] OR “Polymorphisms, Genetic” OR “Gene Polymorphism” OR “Gene Polymorphisms” OR “Polymorphism, Gene” OR “Polymorphisms, Gene” OR “Polymorphism (Genetics) ” OR “Polymorphisms (Genetics) ” OR “Genetic Polymorphisms” OR “Genetic Polymorphism”
#1 AND #2 AND #3
(“Sleep Apnea Syndromes”[MeSH Terms] OR “apnea syndrome sleep”[All Fields] OR (“Sleep Apnea Syndromes”[MeSH Terms] OR (“sleep”[All Fields] AND “apnea”[All Fields] AND “syndromes”[All Fields]) OR “Sleep Apnea Syndromes”[All Fields] OR (“apnea”[All Fields] AND “syndromes”[All Fields] AND “sleep”[All Fields])) OR “Sleep Apnea Syndrome”[All Fields] OR “apnea sleep”[All Fields] OR “apneas sleep”[All Fields] OR “Sleep Apnea”[All Fields] OR “Sleep Apneas”[All Fields] OR “Sleep Hypopnea”[All Fields] OR “hypopnea sleep”[All Fields] OR “hypopneas sleep”[All Fields] OR “Sleep Hypopneas”[All Fields] OR “Hypersomnia with Periodic Respiration”[All Fields] OR (“Sleep Apnea Syndromes”[MeSH Terms] OR (“sleep”[All Fields] AND “apnea”[All Fields] AND “syndromes”[All Fields]) OR “Sleep Apnea Syndromes”[All Fields] OR (“sleep”[All Fields] AND “apnea”[All Fields] AND “mixed”[All Fields] AND “central”[All Fields] AND “obstructive”[All Fields])) OR “Mixed Central and Obstructive Sleep Apnea”[All Fields] OR “sleep apnea mixed”[All Fields] OR “Mixed Sleep Apnea”[All Fields] OR “Mixed Sleep Apneas”[All Fields] OR (“Sleep Apnea Syndromes”[MeSH Terms] OR (“sleep”[All Fields] AND “apnea”[All Fields] AND “syndromes”[All Fields]) OR “Sleep Apnea Syndromes”[All Fields] OR (“sleep”[All Fields] AND “apneas”[All Fields] AND “mixed”[All Fields])) OR “sleep disordered breathing”[All Fields] OR “breathing sleep disordered”[All Fields] OR “sleep disordered breathing”[All Fields] OR “sleep apnea, obstructive”[MeSH Terms] OR “apneas obstructive sleep”[All Fields] OR “Obstructive Sleep Apneas”[All Fields] OR “sleep apneas obstructive”[All Fields] OR “apnea obstructive sleep”[All Fields] OR “Sleep Apnea Hypopnea Syndrome”[All Fields] OR “Obstructive Sleep Apnea Syndrome”[All Fields] OR “Obstructive Sleep Apnea”[All Fields] OR “syndrome obstructive sleep apnea”[All Fields] OR “syndrome sleep apnea obstructive”[All Fields] OR “sleep apnea syndrome obstructive”[All Fields] OR “OSAHS”[All Fields] OR “Upper Airway Resistance Sleep Apnea Syndrome”[All Fields] OR (“sleep apnea, obstructive”[MeSH Terms] OR (“sleep”[All Fields] AND “apnea”[All Fields] AND “obstructive”[All Fields]) OR “Obstructive Sleep Apnea”[All Fields] OR (“syndrome”[All Fields] AND “upper”[All Fields] AND “airway”[All Fields] AND “resistance”[All Fields] AND “sleep”[All Fields] AND “apnea”[All Fields]))) AND (“Racial Groups”[MeSH Terms] OR “group racial”[All Fields] OR “groups racial”[All Fields] OR “Racial Group”[All Fields] OR “Race”[All Fields] OR “Races”[All Fields] OR “Racial Stocks”[All Fields] OR “Racial Stock”[All Fields] OR (“Racial Groups”[MeSH Terms] OR (“racial”[All Fields] AND “groups”[All Fields]) OR “Racial Groups”[All Fields] OR (“stock”[All Fields] AND “racial”[All Fields])) OR (“Racial Groups”[MeSH Terms] OR (“racial”[All Fields] AND “groups”[All Fields]) OR “Racial Groups”[All Fields] OR (“stocks”[All Fields] AND “racial”[All Fields])) OR “Continental Population Groups”[All Fields] OR “Continental Population

Group"[All Fields] OR ("Racial Groups"[MeSH Terms] OR ("racial"[All Fields] AND "groups"[All Fields]) OR "Racial Groups"[All Fields] OR ("group"[All Fields] AND "continental"[All Fields] AND "population"[All Fields])) OR ("Racial Groups"[MeSH Terms] OR ("racial"[All Fields] AND "groups"[All Fields]) OR "Racial Groups"[All Fields] OR ("groups"[All Fields] AND "continental"[All Fields] AND "population"[All Fields])) OR ("Racial Groups"[MeSH Terms] OR ("racial"[All Fields] AND "groups"[All Fields]) OR "Racial Groups"[All Fields] OR ("population"[All Fields] AND "group"[All Fields] AND "continental"[All Fields])) OR ("Racial Groups"[MeSH Terms] OR ("racial"[All Fields] AND "groups"[All Fields]) OR "Racial Groups"[All Fields] OR ("population"[All Fields] AND "groups"[All Fields] AND "continental"[All Fields])) OR "Pedigree"[MeSH Terms] OR "Ethnicity"[MeSH Terms] OR "Nationality"[All Fields] OR "Nationalities"[All Fields] OR "Ethnic Groups"[All Fields] OR "Ethnic Group"[All Fields]) AND ("Genetics"[MeSH Terms] OR "polymorphism, genetic"[MeSH Terms] OR "polymorphisms genetic"[All Fields] OR "Gene Polymorphism"[All Fields] OR "Gene Polymorphisms"[All Fields] OR "polymorphism gene"[All Fields] OR "polymorphisms gene"[All Fields] OR "polymorphism genetics"[All Fields] OR "polymorphisms genetics"[All Fields] OR "Genetic Polymorphisms"[All Fields] OR "Genetic Polymorphism"[All Fields])
```

24 studies

2. **EMBASE Search Strategy:**
```

#3 AND #7 AND #10	
	237
	**#10**
#8 OR #9	
	2,715,851
	**#9**
**'genetic polymorphism'**/exp OR **'genetic polymorphism'**/syn	
	507,073
	**#8**
**'genetics'**/exp OR **'genetics'**/syn	
	2,415,973
	**#7**
#4 OR #5 OR #6	
	753,714
	**#6**
**'ethnicity'**/exp OR **'ethnicity'**/syn	
	202,307
	**#5**
**'race'**/exp OR **'race'**/syn	
	297,766
	**#4**
**'ancestry group'**/exp OR **'ancestry group'**/syn	
	465,910
	**#3**
#1 OR #2	
	116,888
	**#2**
**'sleep apnea, obstructive'**/exp OR **'sleep apnea, obstructive'**/syn	
	67,628
	**#1**
**'sleep apnea syndromes'**/exp OR **'sleep apnea syndromes'**/syn	
	116,445

```

**3. Web of Science Search Strategy:**

```
(Sleep Apnea Syndromes) OR (Sleep Apnea, Obstructive) AND (Racial Groups) OR (Pedigree) OR (Ethnicity) AND (Genetics) OR (Polymorphism, Genetic)
```

22 studies

**3. BVS Search Strategy:**

```
**#1 "Síndromes da Apneia do Sono" OR "Sleep Apnea Syndromes" OR "Síndromes de la Apnea del Sueño"** OR "**Apneia Obstrutiva do Sono" OR "Sleep Apnea, Obstructive" OR "Apnea Obstructiva del Sueño"**
**"Grupos Raciais" OR "Racial Groups" OR "Grupos Raciales" OR "Linhagem" OR "Pedigree" OR "Linaje" OR "Etnicidade" OR "Ethnicity" OR "Etnicidad"**
**"Genética" OR "Genetics" OR" Polimorfismo Genético" OR "Polymorphism, Genetic"**
("Síndromes da Apneia do Sono" OR "Sleep Apnea Syndromes" OR "Síndromes de la Apnea del Sueño" OR "Apneia Obstrutiva do Sono" OR "Sleep Apnea, Obstructive" OR "Apnea Obstructiva del Sueño") AND ("Grupos Raciais" OR "Racial Groups" OR "Grupos Raciales" OR "Linhagem" OR "Pedigree" OR "Linaje" OR "Etnicidade" OR "Ethnicity" OR "Etnicidad") AND ("Genética" OR "Genetics" or" Polimorfismo Genético" OR "Polymorphism, Genetic")
```

444 studies

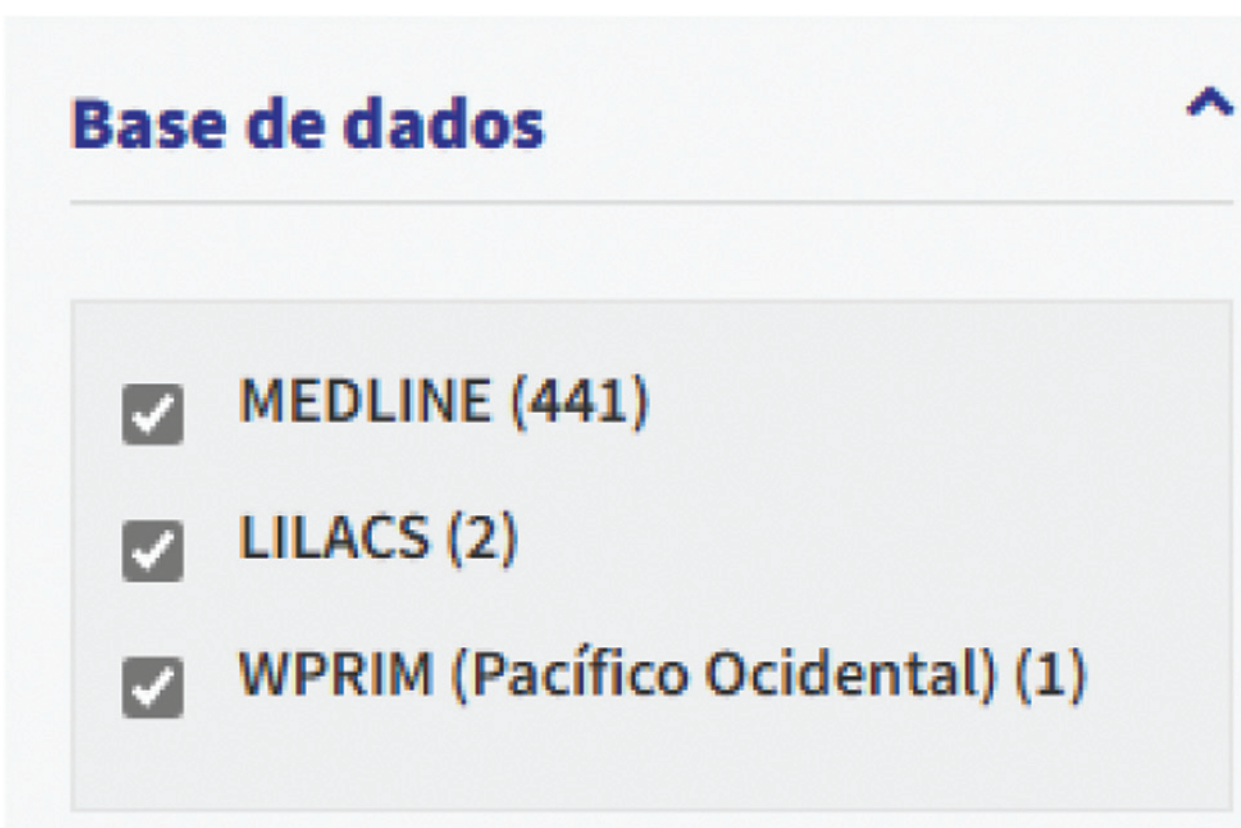
```

5. Cochrane Search Strategy:

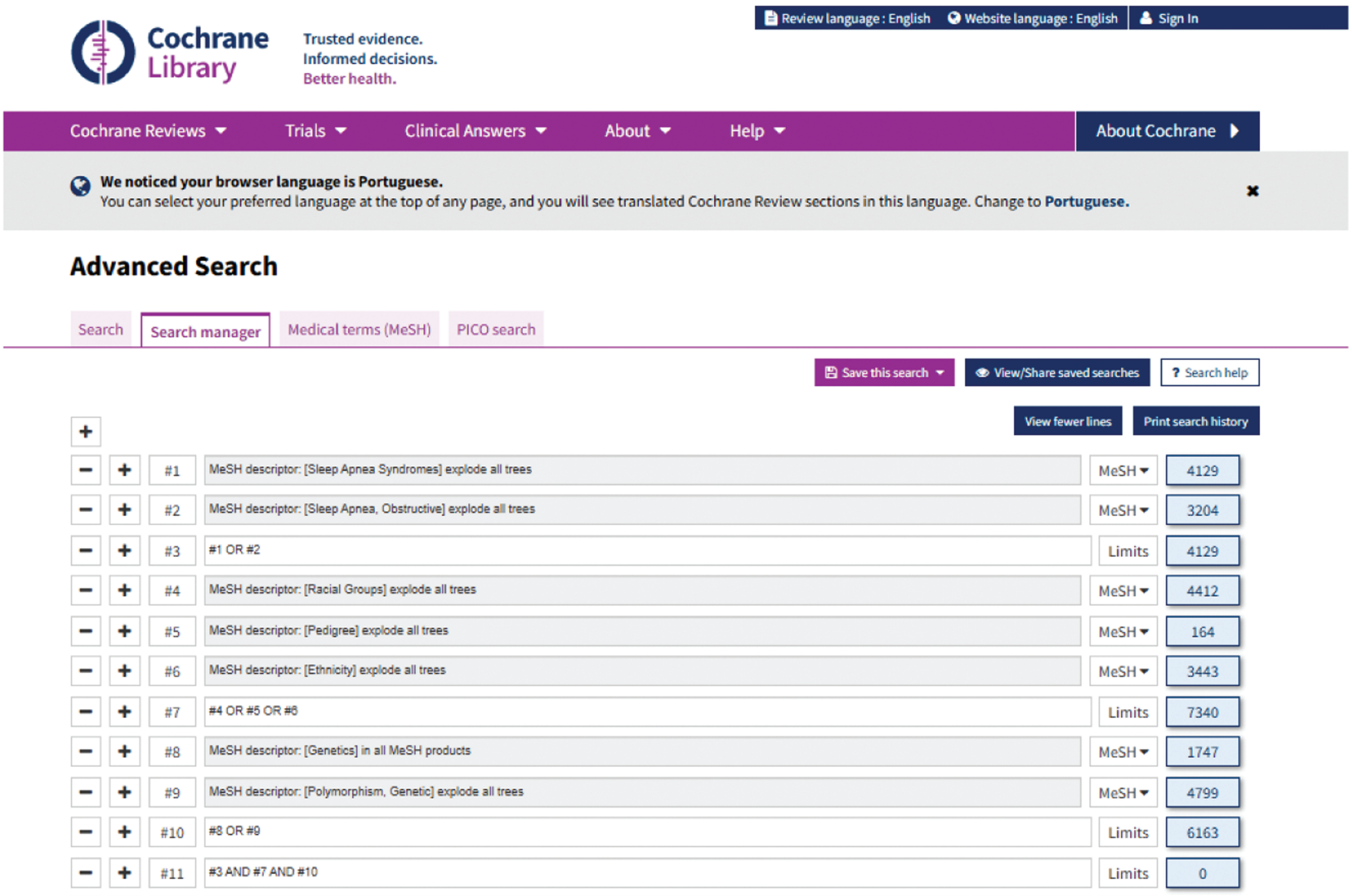

Search Restrictions

- Language: English, Spanish, Portuguese.

Appendix B: Exclusion Reasons

In the present systematic review and meta-analysis, the exclusion reasons used during the study-screening process were based on the inclusion and exclusion criteria established:

Exclusion Reasons

1. **Participants:**
   - Studies involving children or adolescents (under 18 years old). The review focused exclusively on adults with a confirmed diagnosis of obstructive aleep apnea (OSA).
   - Studies with participants without a confirmed diagnosis of OSA. Studies that did not confirm the clinical condition of OSA were excluded.
2. **Interventions:**
   - Interventions not related to the treatment of OSA were excluded. This includes treatments targeting other health conditions without a direct relation to OSA.
   - Studies evaluating interventions that were inappropriate or not recognized for OSA treatment were excluded.
3. **Comparators:**
   - Studies that did not use standard treatment, placebo, or another active intervention as a comparator.
   - Studies that did not present adequate or clearly-defined comparators were also excluded.
4. **Study Designs:**
   - Narrative reviews, systematic reviews, and meta-analyses that had already been published were excluded, as the goal was to conduct an original analysis of primary data.
   - Case reports and case series. Studies that reported only case studies without a control group or comparative analysis were excluded.
   - We included observational studies (case-control, cohort, cross-sectional). We excluded non-primary reports (editorials, letters, reviews) and randomized trials not designed for genetic association.
5. **Other Factors:**
   - Studies that did not segregate data by ethnicity or did not provide genomic information, which hindered the analysis of the impact of ethnicity and molecular markers.
   - Studies in which the primary outcomes were not directly related to the degree of severity or occurrence of OSA in specific populations according to genetic variations.
   - We prespecified qualitative labels for pooled effects: *small* (odd ratio [OR]: 1.00–1.10), *modest* ($OR > 1.10$–1.50), *moderate* ($OR > 1.50$–2.00), and *large* ($OR > 2.00$); analogous thresholds were applied for $OR < 1.00$ by symmetry. The labels are descriptive and do not override confidence intervals. Planned meta-regression (moderators: ancestry, sex, apnea–hypopnea index [AHI] threshold, desaturation criterion, geographic region) was to be performed when ≥ 10 effects were available; this threshold was not met, so no meta-regression was fitted or reported.

- Primary variants: IL6 rs180079520; TNF rs180062910; SLC6A4 5-HTTLPR/STin29; HTR2A rs95262406; LEPR rs379043521 (prespecified from previous literature and data availability). Exploratory variants were summarized without confirmatory testing. Given the small and sparse evidence base, we did not apply multiplicity-adjusted hypothesis testing; effect sizes with 95%CIs are presented and subgroup signals are interpreted as hypothesis-generating.

  These reasons were applied to ensure that the articles included in the systematic review strictly met the eligibility criteria and aligned with the review's objectives of evaluating the influence of ethnicity and molecular markers on the degree of severity of OSA.

 © 2026. The Author(s).
